# The Role of Serum Prolidase Activity, MMP-1, MMP-7, and TGF-β Values in the Prediction of Early Fibrosis in Patients with Moderate to Severe COVID-19

**DOI:** 10.3390/v17070954

**Published:** 2025-07-06

**Authors:** Didem Dogu Zengin, Dilek Ergun, Burcu Yormaz, Recai Ergun, Halil Guven, Muslu Kazim Korez, Halil Ozer, Ali Unlu, Baykal Tulek, Fikret Kanat

**Affiliations:** 1 Department of Pulmonary Medicine, Konya City Hospital, Konya 42020, Turkey; 2 Department of Pulmonary Medicine, Selcuk University, Konya 42250, Turkey; dilekkirbiyik@gmail.com (D.E.); burcyormaz@gmail.com (B.Y.); recaiergun@gmail.com (R.E.); baykaltulek@yahoo.com (B.T.); fkanat@selcuk.edu.tr (F.K.); 3 Department of Biochemistry, Selcuk University, Konya 42250, Turkeyaunlu@selcuk.edu.tr (A.U.); 4 Department of Biostatistics, Selcuk University, Konya 42250, Turkey; mkkorez@gmail.com; 5 Department of Radiology, Selcuk University, Konya 42250, Turkey; drhalilozer@gmail.com

**Keywords:** post-COVID-19, serum prolidase activity, MMP-1, MMP-7, TGF-β, LDH

## Abstract

Background: This study aims to identify predictive factors for pulmonary fibrosis development in COVID-19 patients by analyzing thorax CT (computed tomography) findings, serum prolidase activity, MMP-1, MMP-7, TGF-β values, laboratory findings, and demographic characteristics. Materials and methods: The investigation involved 68 patients, both male and female, aged 18 years and older, who were volunteers and had been diagnosed with confirmed COVID-19. The pulmonologist and the radiologist evaluated the thorax CT by consensus. Patients were evaluated in two categories, group 1 and group 2, based on the status of fibrotic changes, and 3-month fibrosis scores were calculated. Findings in both lungs were calculated and noted for the lobes, considering lobar spread. Correlations between quantitative parameters were assessed with Spearman’s rho correlation coefficient. Comparisons between independent samples were evaluated using either the independent sample *t*-test or the Mann–Whitney U test. We evaluated the relationship between categorical variables using the Pearson chi-square test and Fisher’s exact test. Results: Serum prolidase activity, MMP-1, MMP-7, and TGF-β biomarkers were not statistically significant among groups. LDH was found to be significantly high in the group with fibrotic changes. Additionally, the group with fibrotic changes also had higher levels of fibrinogen. The percentage of neutrophils, the severity of the disease, muscle–joint pain and fatigue symptoms, and the length of hospitalization stay were correlated with the total scores of fibrosis at the third month. In the group with fibrotic changes, the duration of muscle–joint pain and fatigue symptoms and the length of hospitalization were longer than in the other group. Conclusions: The group with fibrotic changes showed an increase in biomarkers. However, this increase did not reach a statistically significant level, suggesting that the third month may be an early period for these changes. The group with fibrotic changes showed high levels of LDH, one of the most important laboratory parameters of pulmonary fibrosis risk factors, along with fibrinogen, suggesting that these parameters are valuable in predicting pulmonary fibrosis. Patients with fibrotic changes can experience specific symptoms, commonly seen in COVID-19.

## 1. Introduction and Purpose

Pneumonia cases with an unknown cause were reported in Wuhan City, Hubei Province, China, on 31 December 2019. Since then, COVID-19 (coronavirus disease) has spread rapidly. WHO (World Health Organization) declared an “International Public Health Emergency” for COVID-19 caused by SARS-CoV-2 (Severe Acute Respiratory Syndrome Coronavirus 2) on 30 January 2020, and a pandemic on 11 March 2020, as a result of its spread to other countries. According to current data, the number of cases worldwide is around 700 million, and the number of confirmed deaths from COVID-19 is approximately 6.5 million [1].

The natural course of patients severely affected by the infection is currently unclear. While some patients will develop pulmonary fibrosis or post-COVID-19 interstitial lung disease (PC-ILD) over time, using our knowledge of infections closely related to the coronavirus pandemic, such as SARS and MERS (Middle East Respiratory Syndrome), we can assume that most patients will stabilize or recover. Given the scale of the pandemic, physicians are likely to encounter large numbers (potentially hundreds of thousands) of patients with PC-ILD, unlike the SARS and MERS epidemics that affected several thousand people [2].

In a study evaluating markers of pulmonary fibrosis in patients with COVID-19 pneumonia using clinical data and thorax CT, researchers found irregular interface, parenchymal bands, and interstitial thickening, evidence of fibrosis in approximately half of the patients on initial CT and in 85–92% of patients on follow-up CT. They suggested that irregular interface and parenchymal bands may be two early markers of pulmonary fibrosis [3].

Biomarkers were also examined to predict pulmonary fibrosis in COVID-19 infection and other diseases. Prolidase, a metalloenzyme among the biomarkers associated with pulmonary fibrosis, is critical for the release of proline in protein metabolism. Although changes in prolidase activity are seen in cancer and many diseases involving fibrotic processes, prolidase activity may be a useful marker in the early detection and monitoring of these diseases [4].

The metalloproteinases MMP-1 (Matrix Metalloproteinase-1) and MMP-7 (Matrix Metalloproteinase-7) are defined as potential peripheral blood biomarkers in idiopathic pulmonary fibrosis (IPF). In a study, MMP-1 and MMP-7 levels were found to be significantly higher in the serum and bronchoalveolar lavage fluid of IPF patients compared to healthy controls [5].

MMP-1/MMP-7 are MMPs known to be significantly expressed in activated alveolar epithelium in IPF lungs. MMP-1, a matrix metalloprotease that mainly degrades fibrillar collagen, is rarely expressed under normal conditions but is highly expressed in reactive alveolar epithelial cells in IPF lungs [6].

In a study, MMP-7 levels were shown to be associated with early fibrotic changes on CT scans of asymptomatic patients with familial interstitial pneumonia [7]. This suggests that MMP-7 may have a profibrotic effect in IPF patients.

When the relationship between TGF-β (transforming growth factor—beta) and pulmonary fibrosis is evaluated, the interaction of the spike protein of SARS viruses with ACE-2 (angiotensin-converting enzyme 2) downregulates the ACE-2 receptor and upregulates TGF-β.

SARS-CoV infection not only increases TGF-β expression but also facilitates signaling activity. ACE-2, a SARS-CoV receptor, is a negative regulator of lung fibrosis. Therefore, SARS-CoV infection may lead to lung fibrosis through multiple signaling activities. TGF-β activation is one of the major contributors to this condition [8].

This study aimed to determine the predictive factors for the development of pulmonary fibrosis by combining thorax CT findings at the time of hospitalization, serum prolidase activity (SPA), MMP-1, MMP-7, TGF-β values, laboratory findings, clinical–radiological features (follow-up thorax CT requested only in the case of clinical necessity) at the 12th week routine clinical controls, and demographic characteristics of patients who were treated for COVID-19 in our respiratory medicine service, had moderate to severe disease, and were discharged.

### Laboratory Evaluation

Research on biochemical/hematological values in the diagnosis, follow-up, and prognosis of COVID-19 patients reveals that these parameters have an important role in predicting the severity of the disease and response to treatment. This has been supported by several studies.

Lymphopenia, one of the leukocyte parameters, was observed in COVID-19 patients, and lymphocyte count was shown to be associated with increased mortality, ARDS, need for intensive care unit follow-up, and severe COVID-19 [9]. The eosinophil count has been thought to be an indicator of response to treatment and recovery in COVID-19.

It was predicted that a high neutrophil count is an indicator of poor prognosis, and when evaluated together with a low lymphocyte count, a high neutrophil/lymphocyte ratio (NLR) can be used as a marker of poor prognosis [10].

Eosinopenia was seen in COVID-19 patients, and the decrease in eosinophils may be explained by a mechanism caused by SARS-CoV-2 inhibiting the release of eosinophils in the bone marrow through glucocorticoid secretion in cases of acute lung injury [11].

D-dimer is elevated in peripheral blood in COVID-19 patients, especially those with severe disease. D-dimer levels are suggested to be associated with disease severity and to be a reliable prognostic marker for mortality in COVID-19 [12].

Thrombocytopenia was observed to be associated with severe disease and mortality in COVID-19 patients and was suggested to be used as a clinical indicator of poor prognosis of the disease [13].

In a cohort study, it was found that COVID-19 patients were more likely to have abnormal PT, aPTT, D-dimer, and fibrinogen levels at hospital admission compared to the control group, and a statistically significant increase in fibrinogen levels was found in COVID-19 patients with ARDS (Acute Respiratory Distress Syndrome) compared to those without ARDS [14].

It was observed that increased levels of ferritin, whose function is to store iron for use in cellular functions, were indicative of a strong inflammatory response in COVID-19, and serum ferritin levels were positively correlated with the severity of COVID-19. Hyperferritinemia was observed in all patients with severe disease at hospital admission [15].

An increase or decrease in LDH (lactate dehydrogenase) was reported to be an indicator of radiographic progression or improvement, and normalization of serum LDH was found to be consistent in predicting treatment success in patients [16].

C-reactive protein (CRP) is a biomarker of systemic inflammation, and high plasma CRP levels were shown to be associated with the severity of COVID-19 pneumonia and longer hospitalization [17].

In adults, high PCT (procalcitonin) was found to be associated with an approximately 5-fold increased risk of severe disease in COVID-19 infection [18].

Multiple studies observed the association of elevated liver enzymes in patients with COVID-19 infection, hypothesizing that viral infection may cause liver damage through direct hepatotoxic injury, drug toxicity, or an immune response-mediated pathway. Acute liver injury and high ALT and AST levels were associated with poor prognosis [19].

In patients with severe COVID-19, significant elevations in renal biomarkers (creatinine, urea) were observed and associated with poor prognosis [20].

## 2. Materials and Methods

### 2.1. Ethical Approval, Study Design, and Inclusion Criteria

This study was derived from the dissertation and was approved by the Selçuk University Faculty of Medicine Clinical Research Local Ethics Committee with the decision numbered 2022/168 on 29 March 2022. This study is a case-cohort study. Patients participating in the study were informed about the study and signed informed consent forms.

The study was conducted between April 2022 and January 2023 in a university hospital that has been determined to be the COVID-19 pandemic center of the province where our hospital is located. Male and female patients aged 18 years and older with a definite diagnosis of COVID-19, who were hospitalized and treated in the Selçuk University Respiratory Medicine Clinic and who volunteered to participate in the study, were included in the study.

### 2.2. Exclusion Criteria for the Study

Pregnant women, patients under 18 years of age, patients with pre-existing interstitial lung disease (ILD), patients with malignancy, and patients who did not sign a consent form were excluded from the study. The study was completed with 68 patients.

All patients were classified according to the 2019 Coronavirus Disease Guidelines (7th edition) published by the National Health Commission of China [21,22,23]. According to the classification, they were grouped as mild, moderate, severe, and critical types.

### 2.3. Evaluation of Radiology Images

Thorax CT images of the patients, requested only in the case of clinical necessity at the 12th-week routine check-ups in our clinic after discharge, were included in the study. The thorax CT images of the patients were evaluated in a single session by the consensus of a pulmonologist and a radiologist. CT scans were evaluated together by an experienced specialist radiologist and a professor of pulmonology medicine, and a decision for fibrosis was made meticulously with a common consensus.

Traction bronchiectasis or honeycomb appearance was considered an indicator of significant fibrosis (also the findings associated with long-term fibrosis [24]), and the patients were divided into two groups. The group with significant fibrotic changes, such as traction bronchiectasis or honeycombing, was group 1, and the group without significant fibrotic changes was group 2.

The third-month total fibrosis score was obtained by evaluating reticulation and lobar spread of the subpleural band, traction bronchiectasis, or the existence of honeycomb formation.

### 2.4. Evaluation of Laboratory Results and Fibrosis Score

The laboratory results obtained during hospitalization were utilized. The levels of serum prolidase activity, MMP-1, MMP-7, and TGF-β were analyzed using ELISA kits.

The identified observations in both lungs were evaluated and assigned scores, taking into account lobar spread, with a score of 0–5 for the lobes and a maximum score of 25 points. The third-month total fibrosis score was calculated by summing individual lobe scores, with 1 point <5%, 2 points 5–25%, 3 points 26–49%, 4 points 50–75%, and 5 points >75%.

### 2.5. Statistical Analysis and Determination of Sample Size

In order to compare the serum prolidase, MMP-1, MMP-7, and TGF-β concentrations of patients diagnosed with moderate to severe COVID-19 disease, this study utilized a calculated sample size to determine the difference between patients who developed early fibrosis and those who did not develop fibrosis. For this purpose, it was planned to include at least 68 patients in the study, with at least 34 patients in each group, in order to reach 90% statistical power with a 5% significance level and an effect size of 0.8. The “pwr” package in R version 3.6.0 (The R Foundation for Statistical Computing, Vienna, Austria) was used to calculate the sample size.

All statistical analyses were performed with the help of R version 3.6.0 software (The R Foundation for Statistical Computing, Vienna, Austria). Before the analyses, the normality of the data was confirmed with the help of Shapiro–Wilk’s normality test and Q-Q graphs, and the homogeneity assumptions of group variances were evaluated with the Levene test. The statistics for descriptive numerical variables in the data are presented as the mean and standard deviation (minimum–maximum) and the median (interquartile range [IQR]: 1st quarter–3rd quarter). The categorical variables were presented as frequency (*n*) and percentage (%). The relationship between the fibrosis total score in the third month and several variables, including the patients’ age, smoking (pack/years), BMI, initial blood parameters, and serum levels of prolidase activity, MMP-1, MMP-7, and TGF-β, was assessed using Spearman’s rho correlation coefficient. The relationships between the presence of comorbidities and symptoms and the 3-month fibrosis score of the patients were compared using the independent samples t-test and Mann–Whitney U test. The groups with and without fibrotic change were compared using several statistical techniques, including the independent samples t-test, Mann–Whitney U test, Pearson chi-square test, Yates continuity corrected chi-square test, and Fisher’s exact test.

## 3. Results

The investigation was conducted with a total of 68 patients; 39 of the patients were male (57.4%) and 29 were female (42.6%). The median age was 65.5 (29–86). The measurement results of biomarkers and the demographic characteristics, classification, comorbidities, oxygen therapy, symptoms, and biomarkers are given in Table 1.

A significant positive relationship was observed between the length of hospital stay and the fibrosis total score at the third month (Spearman’s *rho* = 0.461, *p <* 0.001). There was a significant positive correlation between the neutrophil percentages and the third-month fibrosis total scores (Spearman’s rho = 0.249, *p* = 0.040). However, at the time of admission, there was no significant correlation observed between the blood values (biomarkers) and the third-month fibrosis total score (*p* > 0.05). There was no significant correlation observed between the third-month fibrosis total score and other quantitative parameters (Table 2).

The relationship between the moderate and severe patient groups and the third-month fibrosis total score is summarized in Table 3. Patients were categorized based on COVID-19 classification, and it was observed that the fibrosis total score at the third month was significantly higher in the severe group compared to the moderate group (6.50 [0–16] vs. 3.0 [0–13], *p* = 0.019).

The relationships between the third-month fibrosis total score, comorbidities, and symptoms are summarized in Table 4. Upon evaluating the patients’ comorbidities, it was observed that there was no statistically significant difference between the groups with and without comorbidities regarding fibrosis total scores at the third month (*p >* 0.05).

Patients experiencing muscle–joint pain symptoms had a higher fibrosis total score in the third month compared to those without these symptoms (7 [0–16] vs. 5 [0–16], *p* = 0.030). Patients experiencing fatigue symptoms had a significantly higher fibrosis overall score in the third month compared to participants without these symptoms (7 [0–16] vs. 5 [0–11], *p* = 0.010).

No significant relationship was observed between the most common symptoms in patients (cough and fever) and the third-month fibrosis total score (*p* > 0.05).

**Figure 1:**
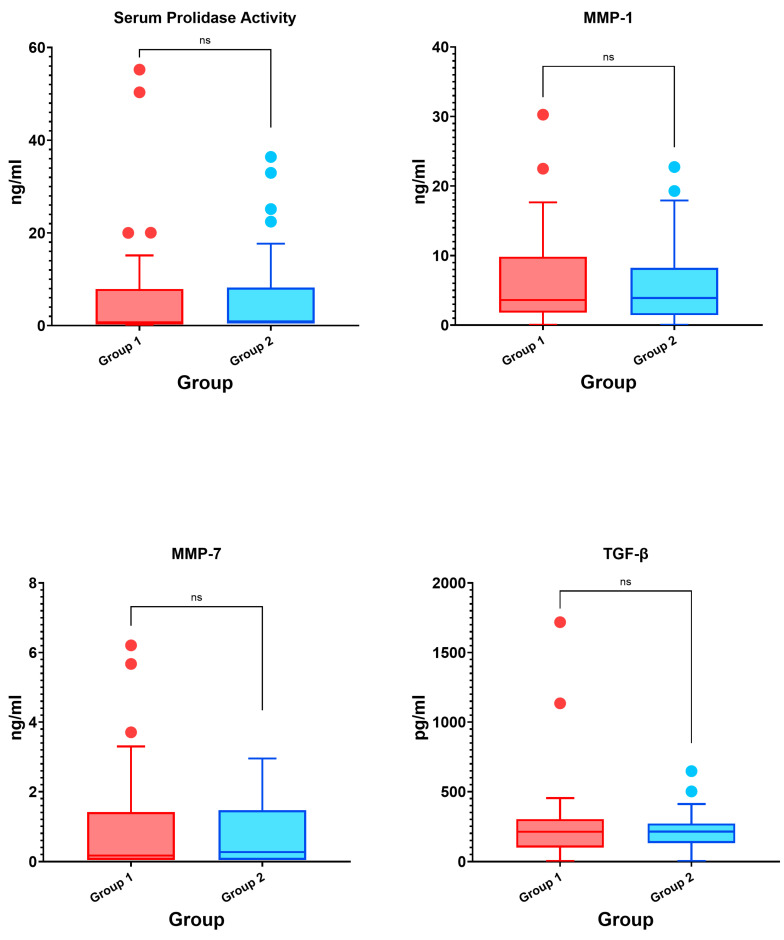
Box plot graphs of the intergroup comparison of biomarkers (ns: non-significant).

Demographic characteristics, smoking history, biomarkers, blood parameters at the time of admission, and their relationships with clinical characteristics of group 1 and group 2 patients are summarized in Table 5 and Figure 1. There was no significant difference between the groups in terms of age and BMI (*p* > 0.05). There was no statistically significant difference between the groups in terms of smoking and smoking exposure (secondhand smoker) (*p* = 0.584 and *p* = 0.631, respectively).

Although the results revealed higher levels of serum prolidase activity (0.90 [0.50–7.30] vs. 0.50 [0.50–7.27], *p* = 0.491), serum MMP-1 level (3.92 [1.47–7.63] vs. 3.62 [1.82–9.28], *p* = 0.956), serum MMP-7 level (0.27 [0.05–1.41] vs. 0.17 [0.05–1.36], *p* = 0.927), and serum TGF-β level (213.35 [140.25–263.65] vs. 211.57 [107.88–295.81], *p* = 0.966) in the group with fibrotic changes compared to the group without fibrotic changes, these differences were not statistically significant.

LDH levels in group 1 were significantly higher compared to group 2 (363 [290.25–472.25] vs. 272.5 [215.25–360.25], *p* = 0.012). Additionally, the fibrinogen values at the time of admission in group 1 were also significantly higher than in group 2 (562.50 [452.50–714.74] vs. 445.5 [368.75–538.0], *p* = 0.002). There was no significant difference between the groups for other blood parameters at the time of admission (*p* > 0.05).

The results demonstrated that the length of hospitalization was greater in group 1 compared to group 2 (11 [10–16] vs. 8.5 [5–10.75], *p* <  0.001).

Comparisons of group 1 and group 2 patients in terms of gender, classification, comorbidities, and symptoms are summarized in Table 6. Upon evaluating group 1 and group 2 based on gender and classification (disease severity), no statistically significant difference was observed between the groups (*p* > 0.05). There was no significant difference between groups in terms of comorbidities (*p* > 0.05). When both groups were compared in terms of symptoms, muscle–joint pain and fatigue were more common in group 1 (*p* = 0.015 and *p* = 0.029, respectively). There was no statistically significant difference observed among the groups in terms of other symptoms (*p* > 0.05).

A comparison of group 1 and group 2 patients in terms of oxygen treatments is summarized in Table 7. No statistically significant difference was observed between group 1 and group 2 in terms of oxygen treatment (*p* > 0.05).

## 4. Discussion

Prolidase is essential for the construction and degradation of collagen-containing connective tissue, and changes in its activity are present in many diseases involving fibrotic processes [4]. In a study evaluating idiopathic and ischemic dilated cardiomyopathies, which exhibited the same histopathological feature of fibrosis, serum prolidase activity was found to be significantly lower in both groups compared to healthy volunteers [25].

In a study investigating patients with chronic obstructive pulmonary disease (COPD), an inflammatory lung disease in which irreversible fibrotic changes occur in the airways, lower plasma prolidase activity was observed in COPD patients compared to the control group (*p* < 0.05) [26]. In our study, no significant relationship was observed between the third-month total fibrosis score and serum prolidase levels. When evaluated in terms of fibrotic changes, although there was no significant difference between the groups, they were observed to be higher in the fibrotic group compared to the other group at the beginning. This can also be explained by the fact that it is an early period for changes in prolidase activity during the fibrosis process. Significant changes may be found when evaluated in the future.

In a recent study, MMP1 and MMP7, which are identified as potential peripheral blood biomarkers in IPF, were found to be significantly higher in serum and bronchoalveolar lavage fluid in IPF patients compared to healthy controls [5]. In a different study, MMP-7 levels were associated with early fibrotic changes on the CT scans of asymptomatic patients with familial interstitial pneumonia [7].

Our study has some limitations. For example, the patients considered in this study who were infected with COVID-19 may have been at different stages of the disease at hospital admission and hospitalization. Although the biomarkers included in our main hypothesis were found to be higher in the fibrotic group, no significant statistical difference was observed.

Studies conducted for COVID-19 showed that males have higher mortality or severe infection rates than females. While some studies reported a higher incidence of COVID-19 infection in males, some studies found no difference in the incidence of COVID-19 infection [27].

In a study involving 72,314 suspected or confirmed COVID-19 cases in China, it was found that 51.4% of the patients were male, with 22,981 cases, and mortality was significantly higher in males than females [28].

In a study conducted with 14,712 patients with COVID-19, the all-cause mortality rate was higher in males than females (*p* < 0.001) [29]. In our study, the number of male patients (*n* = 39) was high among patients with COVID-19 infection, and the proportion of male patients (*n* = 33) was high in the severe disease group.

In a meta-analysis of 2018 patients evaluating pulmonary fibrosis after COVID-19, the mean age of fibrotic patients was 59 years, while the mean age of non-fibrotic patients was 48.5 years (*p* = 0.003), and there was no significant difference between fibrotic and non-fibrotic patients in terms of BMI (25.23 and 24.75, respectively), evaluated by only two studies, and no significant gender difference (53.8% male and 46.2% female) was observed between groups [30].

In our study, age and body mass index were not significantly associated with the third-month fibrosis total score. There was no significant difference between group 1 and group 2 in terms of age and body mass index, and no significant difference was found in terms of gender (*p* > 0.05).

Although the symptoms of patients at hospital admission differ in COVID-19, the common symptoms are similar in most studies.

In a study evaluating the data of 1099 COVID-19 patients, fever was present in 43.8% of the patients during hospital admission and developed in 88.7% of the patients at hospitalization, and the second-most common symptom was a cough (67.8%), followed by nausea or vomiting (5.0%) and diarrhea (3.8%) [31].

In one of the first studies on COVID-19, common symptoms at the onset of the disease were fever (98%), cough (76%), and myalgia or fatigue (44%), with less common symptoms being phlegm (28%), headache (8%), and diarrhea (3%). It was observed that 22 of 40 patients (55%) developed dyspnea [32].

In a study including 10 studies and a total of 1995 cases, clinical symptoms of COVID-19 patients were fever (88.5%), cough (68.6%), myalgia/fatigue (35.8%), phlegm (28.2%), and shortness of breath (21.9%) [33]. According to a meta-analysis, among COVID-19 symptoms, cough (47.4%), chest pain (27.6%), and fever (72.4%) were more common in the fibrotic group compared to the non-fibrotic group (*p* < 0.05) [30].

In our study, the most common symptoms at hospital admission were cough (57.4%), fever (52.9%), and fatigue (52.9%). Our patients frequently displayed cough and fever symptoms, in line with the literature.

In a study of 80 COVID-19 patients, the frequencies of myalgia and fatigue were 46.1% and 50%, respectively. Laboratory data showed a significant increase in creatinine kinase (CK) level and lymphocyte count in patients with myalgia symptoms (*p* < 0.05). It was reported that caution should be exercised regarding muscle damage due to increased CK in these patients and that extremely high values may indicate a progressive myopathic process [34]. In our study, when the relationship between the symptoms at hospital admission and the third-month fibrosis total score was evaluated, the score was higher in patients with muscle–joint pain symptoms compared to the group without symptoms (*p* = 0.031). The third-month fibrosis total score of patients with fatigue symptoms was found to be significantly higher than that of the group without symptoms (*p* = 0.006). When groups 1 and 2 were compared in terms of symptoms, muscle–joint pain (*p* = 0.015) and fatigue symptoms were more common in group 1 (*p* = 0.029). There was no difference in other symptoms (*p* > 0.05).

In terms of clinical presentation, COVID-19 pneumonia was more severe in some patient groups, and many studies on this subject demonstrated that comorbidities play a role in this situation. In a study evaluating 191 COVID-19 patients, hypertension was found to be the most common comorbidity, followed by diabetes mellitus and coronary heart disease [35].

In a study evaluating 654 COVID-19 patients in our country, it was observed that the most common comorbidities were hypertension, diabetes mellitus, and cardiovascular diseases (25.3%, 12.4%, and 10.7%, respectively) [36].

In a study in which 81 patients were evaluated for pulmonary fibrosis, the fibrosis group had higher rates of DM, hypertension, chronic lung disease, chronic liver disease, and cardiovascular and cerebrovascular diseases than the non-fibrosis group. Abnormal parameters, including leukocytosis, neutrophilia, lymphopenia, eosinopenia, high CRP, and d-dimer, were found to be significantly different between the groups [37]. In our study, the most common comorbidities were noted as DM (44.1%, 30), respiratory disease (42.6%, 29), and hypertension (41.2%, 28). No significant difference was observed between the groups in terms of comorbidities (*p* > 0.05). There was also no significant correlation between comorbidities and the third-month fibrosis total score (*p* > 0.05).

A study conducted for SARS showed a positive correlation between peak LDH level and pulmonary fibrosis at hospital admission [38]. In our study, LDH values at hospital admission in the group with fibrotic changes were significantly higher than in the group without fibrotic changes (*p* = 0.012). The fibrinogen values were also significantly higher in group 1 compared to group 2 at hospital admission (*p* = 0.002). In light of these data, LDH values were noted to be high in the fibrotic group at hospital admission, in line with the literature.

In a study evaluating 227 COVID-19 patients, pulmonary fibrosis was found to be significantly associated with some coagulation indices and fibrinogen [39]. Similarly, in our study, it was observed that the fibrinogen value in blood parameters at hospital admission was higher in group 1 than in group 2 (*p* = 0.008).

Huang et al. indicated that neutrophil, neutrophil-to-lymphocyte ratio (NLR), C-reactive protein (CRP), and LDH levels were significantly above the normal range during the first four weeks after hospitalization in patients with extensive fibrosis at two-month follow-up. Conversely, CRP and LDH were shown to increase within two weeks but then sharply decrease to normal levels in the non-fibrosis group [37]. In our study, there was a significant and positive relationship between neutrophil percentages and the third-month fibrosis total scores (*p* = 0.040).

In another study evaluating pulmonary fibrosis after COVID-19, some predictive factors, such as old age and smoking, were reported. The incidence of post-pulmonary fibrosis was found to be much higher in patients with a smoking history than in non-smokers, and post-pulmonary fibrosis was observed to develop in 18 out of 30 smokers [40]. In our study, no significant relationship was found between smoking exposure (secondhand smoker) (*p* = 0.793) and the third-month fibrosis total score. There was no difference between the groups in terms of smoking and smoking exposure (secondhand smoker) (*p* = 0.584), and our study was different in this respect.

A significant and positive relationship was detected between the patients’ length of hospitalization (*p* < 0.001) and the third-month fibrosis total score. It was observed that the length of hospitalization was longer in group 1 compared to group 2 (*p* < 0.001). In a study in which fibrotic and non-fibrotic groups were compared after COVID-19, a statistically significant difference was observed between both groups in terms of ICU hospitalization and total length of hospitalization (*p* ˂ 0.001). On the other hand, the length of hospitalization in the fibrotic group was found to be longer than in the non-fibrotic group (23.26 ± 20.89 vs. 8.56 ± 7.03 days) [41]. The data in the relevant study was found to support our results.

Our study’s single-center design and small sample size are some of its limitations. Additionally, the fact that patients may have had different disease stages during admission may have affected our results.

## 5. Conclusions

In our study investigating the role of serum prolidase activity, MMP-1, MMP-7, and TGF-β values in predicting early fibrosis, it was observed that these biomarkers were increased in the group with fibrotic changes, although not at a statistically significant level. LDH, one of the most important laboratory parameters of pulmonary fibrosis, and fibrinogen were high in the group with fibrotic changes. Neutrophil percentages, disease severity, muscle–joint pain, fatigue symptoms, and length of hospital stay were found to be associated with 3-month fibrosis total scores. Musculoskeletal pain, fatigue symptoms, and length of hospital stays were higher in the fibrotic group compared to the group without fibrotic changes.

Based on our findings, we believe that additional studies are required to accurately predict pulmonary fibrosis following COVID-19 and evaluate the long-term outcomes of the disease.

## Figures and Tables

**Table 1:** Summary of the overall statistical distribution of patients’ quantitative and qualitative findings.

	Patient (*n* = 68)
Demographic characteristics	
Age (year)	66.5 (29–86)
Gender (male/female)	39 (57.4)/29 (42.6)
BMI (kg/m^2^)	27.9 (15.3–50.8)
Length of hospitalization (days)	10 (4–30)
Smoking	
Ex-smoker	12 (17.6)
Non-smoker	52 (76.5)
Smoker	4 (5.9)
Smoking (pack/years)	45 (5–141)
Classification	
Moderate	14 (20.6)
Male	6 (42.9)
Female	8 (57.1)
Severe	54 (79.4)
Male	33 (61.1)
Female	21 (38.9)
Comorbidity ^†^	
HT	28 (41.2)
DM	30 (44.1)
Chronic pulmonary diseases ^ǂ^	29 (42.6)
CAD	17 (25)
HL	5 (7.4)
CHF	4 (4.4)
Thyroid disorder	5 (7.4)
Rheumatologic	1 (1.5)
Other ^§^	7 (10.3)
Symptoms ^†^	
Fever	36 (52.9)
Cough	39 (57.4)
Dyspnea	30 (44.1)
Muscle–joint pain (myalgia)	31 (45.6)
Sputum	5 (7.1)
Headache	4 (5.9)
Sore throat	12 (17.6)
Diarrhea	6 (8.8)
Nausea	8 (11.8)
Vomiting	6 (8.8)
Abdominal pain	1 (1.5)
Chest pain	1 (1.5)
Loss of taste and smell	6 (8.8)
Fatigue	36 (52.9)
Biomarkers	
Serum prolidase activity (ng/mL)	0.50 (0.063–55.22)
MMP-1 (ng/mL)	3.91 (0.05–30.27)
MMP-7 (ng/mL)	0.172 (0.050–6.20)
TGF-β (pg/mL)	212.42 (5.0–1718.29)
Oxygen therapy ^†^	
No oxygen support (room air)	15 (22.1)
Supplemental oxygen therapy (nasal cannula)	50 (73.5)
High flow nasal oxygen therapy	2 (2.9)
NIMV	5 (7.4)

Data are presented as interquartile range (median [minimum–maximum]) or frequency (*n*) and percentage (%). **^†^** Multiple features may be present in a patient. ^ǂ^ Asthma, chronic obstructive pulmonary disease. ^§^ Neurological, gastrointestinal, renal, and liver disorders. Abbreviations: HT = hypertension; DM = diabetes mellitus; CAD = coronary artery disease; HL = hyperlipidemia; CHF = congestive heart failure.

**Table 2:** Relationships between the third-month fibrosis total score and the demographic and clinical characteristics of the patients, smoking exposure, biomarkers, and blood parameters at the time of hospitalization.

	THIRD-MONTH FIBROSIS TOTAL SCORE	
	Spearman’s *rho*	*p*-Value
Demographic characteristics		
Age (years)	0.104	0.401
Smoking (pack/years)	−0.032	0.793
BMI (kg/m^2^)	−0.169	0.169
Clinical characteristics		
Length of hospitalization (days)	0.461	<0.001
Biomarkers		
Serum prolidase activity (ng/mL)	0.051	0.677
MMP-1 (ng/mL)	0.012	0.925
MMP-7 (ng/mL)	−0.078	0.525
TGF-β (pg/mL)	−0.034	0.784
BLOOD PARAMETERS AT THE TIME OF HOSPITALIZATON		
WBC	0.219	0.073
HGB	−0.067	0.587
HCT	−0.090	0.465
PLT	0.185	0.131
MCV	0.024	0.848
RDW	0.003	0.981
RBC	−0.066	0.593
PDW	−0.008	0.947
MPV	−0.124	0.313
NEUTROPHIL	0.191	0.119
NEUTROPHIL (%)	0.249	0.040
LYMPHOCYTE	−0.132	0.285
LYMPHOCYTE (%)	−0.196	0.110
LDH	0.194	0.113
FIBRINOGENE	0.209	0.087
FERRITIN	0.046	0.709
D-DIMER	0.001	0.996
CRP	0.115	0.350
PROCALCITONIN	0.007	0.952
CREATININE	0.053	0.666
UREA	0.113	0.358
ALT	−0.011	0.928
AST	0.077	0.533

**Table 3:** Relationships between the third-month fibrosis total score and moderate and severe patient groups.

Clinical Group	THIRD-MONTH FIBROSIS TOTAL SCORE	*p*-Value
Moderate (*n* = 14)	3.0 (0–13)	0.019 *
Severe (*n* = 54)	6.50 (0–16)	

Data are presented as interquartile range (median [minimum–maximum]). * Mann–Whitney U test.

**Table 4:** Relationships between third-month fibrosis total score, comorbidities, and symptoms.

	THIRD-MONTH FIBROSIS TOTAL SCORE		*p*-Value
COMORBIDITY	No	Yes	
HT	6 (0–16)	7 (0–16)	0.531
DM	7 (0–16)	6 (0–16)	0.833
Chronic pulmonary diseases ^ǂ^	7 (0–16)	6 (0–16)	0.727
CAD	6 (0–16)	7 (0–16)	0.373
HL	6 (0–16)	6 (0–16)	0.767
SYMPTOMS			
Fever	6 (0–16)	6 (0–16)	0.863
Cough	7 (0–13)	6 (0–16)	0.746
Dyspnea	6 (0–13)	7 (0–16)	0.203
Muscle–joint pain (myalgia)	5 (0–16)	7 (0–16)	0.030
Sputum	6 (0–16)	4 (2–11)	0.715
Headache	6 (0–16)	4 (1–11)	0.660
Sore throat	6 (0–16)	6 (0–11)	0.324
Diarrhea	6 (0–16)	6 (0–9)	0.841
Nausea	6 (0–16)	7 (0–12)	0.491
Vomiting	6 (0–16)	8 (5–11)	0.264
Loss of taste and smell	6 (0–16)	6 (0–11)	0.958
Fatigue	5 (0–11)	7 (0–16)	0.010

Data are presented as interquartile range (median [minimum–maximum]). ^ǂ^ Asthma, chronic obstructive pulmonary disease. Abbreviations: HT = hypertension; DM = diabetes mellitus; CAD = coronary artery disease; HL = hyperlipidemia.

**Table 5:** Comparing group 1 and group 2 in terms of the relationship between demographic characteristics, smoking history, biomarkers, blood parameters at the time of admission, and clinical characteristics.

	GROUP 1 (*n* = 34) With Fibrotic Changes	GROUP 2 (*n* = 34) Without Fibrotic Changes	*p*-Value
DEMOGRAPHIC CHARACTERISTICS			
Age (years)	67 (60.75–73)	66 (60.25–73.75)	0.825
BMI (kg/m^2^)	27.90 (26.10–32.22)	28.40 (25.47–31.80)	0.932
SMOKING (EX/NON/SMOKER)	6/25/3	6/27/1	0.584
SECONDHAND SMOKE (EXPOSURE)	40 (15–141)	50 (5–80)	0.631
BIOMARKERS			
Serum prolidase activity (ng/mL)	0.90 (0.50–7.30)	0.50 (0.50–7.27)	0.491
MMP-1 (ng/mL)	3.92 (1.47–7.63)	3.62 (1.82–9.28)	0.956
MMP-7 (ng/mL)	0.27 (0.05–1.41)	0.17 (0.05–1.36)	0.927
TGF-β (pg/mL)	213.35 (140.25–263.65)	211.57 (107.88–295.81)	0.966
BLOOD PARAMETERS AT THE TIME OF HOSPITALIZATON			
WBC	9.63 (6.36–11.73)	7.61 (5.92–9.69)	0.232
HGB	13.13 ± 2.26	13.04 ± 2.12	0.864
HCT	40.30 ± 6.64	40.43 ± 6.83	0.938
PLT	235.50 (172.75–293.75)	181 (136.75–236)	0.078
MCV	87.8 (82.15–90.47)	86.65 (83.53–88.95)	0.540
RDW	14.30 (13.60–16.13)	14.30 (13.60–15.20)	0.811
RBC	4.77 (4.35–5.03)	4.54 (4.25–5.16)	0.768
PDW	13.55 (11.93–16.60)	13.45 (11.60–16.70)	0.864
MPV	10.15 (8.22–10.70)	10.30 (9.40–11.10)	0.117
NEUTROPHIL	7.27 (4.19–9.94)	4.85 (3.64–8.17)	0.332
NEUTROPHIL (%)	79.05 (66.50–87.97)	76.60 (61.32–81.95)	0.185
LYMPHOCYTE	0.95 (0.75–1.65)	1.10 (0.81–1.66)	0.524
LYMPHOCYTE (%)	12.65 (7.26–22.60)	15.15 (10.10–26.38)	0.473
LDH	363 (290.25–472.25)	272.5 (215.25–360.25)	0.012
FIBRINOGENE	562.50 (452.50–714.74)	445.5 (368.75–538.0)	0.002
FERRITIN	425.35 (114.75–769)	273.5 (148.5–389.25)	0.251
D-DIMER	463.50 (252.25–802.75)	512.50 (305.5–830)	0.496
CRP	88.15 (23.38–144.75)	75.15 (14.20–105)	0.253
PROCALCITONIN	0.11 (0.05–0.22)	0.10 (0.07–0.14)	0.859
CREATININE	0.95 (0.81–1.30)	0.98 (0.88–1.15)	0.677
UREA	36.20 (32.20–53.75)	40.50 (29.90–53.65)	0.917
ALT	20.75 (15.18–26.50)	19 (15.22–33.75)	0.888
AST	27 (21.40–38.75)	22.80 (17.23–34.0)	0.303
NLR	6.94 (3.16–11.69)	4.77 (2.28–8.33)	0.308
CLINICAL CHARACTERISTICS			
Length of hospitalization (days)	11 (10–16)	8.5 (5–10.75)	0.001

Data are presented as mean ± SD or interquartile range (median [minimum–maximum]).

**Table 6:** Comparison of group 1 and group 2 patients in terms of gender, classification, comorbidities, and symptoms.

	GROUP 1 (*n* = 34) With Fibrotic Changes	GROUP 2 (*n* = 34) Without Fibrotic Changes	Overall	*p*-Value
Gender				
Female	13 (38.2%)	16 (47.1%)	29 (42.6%)	0.624
Male	21 (61.8%)	18 (52.9%)	39 (57.4%)	
Classification				
Moderate	4 (11.8%)	10 (29.4%)	14 (20.6%)	0.134
Severe	30 (88.2%)	24 (70.6%)	54 (79.4%)	
COMORBIDITIES ^†^				
HT	16 (47.1%)	12 (35.3%)	28 (41.2%)	0.460
DM	13 (38.2%)	17 (50%)	30 (44.1%)	0.464
Chronic pulmonary diseases ^ǂ^	15 (44.1%)	14 (41.2%)	29 (42.6%)	>0.999
CAD	12 (35.3%)	5 (14.7%)	17 (25%)	0.093
SYMPTOMS ^†^				
Fever	21 (61.8%)	15 (44.1%)	36 (52.9%)	0.224
Cough	19 (55.9%)	20 (58.8%)	39 (57.4%)	>0.999
Dyspnea	18 (52.9%)	12 (35.3%)	30 (44.1%)	0.222
Muscle–joint pain	21 (61.8%)	10 (29.4%)	31 (45.6%)	0.015
Sputum	1 (2.9%)	4 (11.8%)	5 (7.4%)	0.356
Headache	1 (2.9%)	3 (8.8%)	4 (5.9%)	0.614
Sore throat	4 (11.8%)	8 (23.5%)	12 (17.6%)	0.340
Diarrhea	2 (5.9%)	4 (11.8%)	6 (8.8%)	0.673
Nausea	5 (14.7%)	3 (8.8%)	8 (11.8%)	0.709
Vomiting	4 (11.8%)	2 (5.9%)	6 (8.8%)	0.673
Adbominal pain	0 (0%)	1 (2.9%)	1 (1.5%)	>0.999
Loss of taste and smell	3 (8.8%)	3 (8.8%)	6 (8.8%)	>0.999
Fatigue	23 (67.6%)	13 (38.2%)	36 (52.9%)	0.029

Data are presented as frequency (*n*) and percentage (%). ^†^ Multiple features may be present in a patient. ^ǂ^ Asthma, chronic obstructive pulmonary disease. Abbreviations: HT = hypertension; DM = diabetes mellitus; CAD = coronary artery disease.

**Table 7:** Comparison of group 1 and group 2 patients in terms of oxygen therapy.

OXYGEN THERAPY	GROUP 1 (*n* = 34) With Fibrotic Changes	GROUP 2 (*n* = 34) Without Fibrotic Changes	Overall	*p*-Value
Patients followed in room air	4 (11.8%)	10 (29.4%)	14 (20.6%)	0.134
Patients requiring supplemental oxygen therapy (non-room air)	30 (88.2%)	24 (70.6%)	54 (79.4%)	
With nasal oxygen cannula	28 (82.4%)	22 (64.7%)	50 (73.5%)	0.169
Without nasal oxygen cannula	6 (17.6%)	12 (35.3%)	18 (26.5%)	
With high-flow oxygen therapy	1 (2.9%)	1 (2.9%)	2 (2.9%)	>0.999
Without high-flow oxygen therapy	33 (97.1%)	33 (97.1%)	66 (97.1%)	
With NIMV	3 (8.8%)	2 (5.9%)	5 (7.4%)	>0.999
Without NIMV	31 (91.2%)	32 (94.1%)	63 (92.6%)	

Data are presented as frequency (*n*) and percentage (%).

## Data Availability

The raw data supporting the conclusions of this article will be made available by the authors on request.

## Abbreviations

CT: Computed tomography; WHO: World Health Organization; COVID-19: Coronavirus disease-19; SARS-CoV-2: Severe Acute Respiratory Syndrome Coronavirus 2; CoV: Coronavirus; MERS: Middle East Respiratory Syndrome; SARS: Severe Acute Respiratory Syndrome; IPF: Idiopathic pulmonary fibrosis; ACE: Angiotensin-converting enzyme; ELISA: Enzyme-linked immunosorbent assay; MMP: Matrix Metalloproteinase; MMP-1: Matrix Metalloproteinase-1; MMP-7: Matrix Metalloproteinase-7; TGF-β: Transforming growth factor—beta; WBC: White blood cell; LDH: Lactate dehydrogenase; CRP: C-reactive protein; ALT: Alanine transaminase; AST: Aspartate transaminase; HGB: Hemoglobin; HCT: Hematocrit; PLT: Platelet; RBC: Red blood cell; MCV: Mean corpuscular volume; MCH: Mean corpuscular hemoglobin; RDW: Red cell distribution width; MPV: Mean platelet volume; PDW: Platelet distribution width; DM: Diabetes mellitus; HT: Hypertension; RNA: Ribonucleic acid; ICU: Intensive care unit; ARDS: Acute Respiratory Distress Syndrome; APTT: Activated partial thromboplastin time; PT: Prothrombin time; NLR: Neutrophil to lymphocyte ratio; BMI: Body mass index; CAD: Coronary artery disease; PC-ILD: post-COVID-19 interstitial lung disease; SD: Standard deviation.
